# Amplified Host Defense by Toll-Like Receptor-Mediated Downregulation of the Glucocorticoid-Induced Leucine Zipper (GILZ) in Macrophages

**DOI:** 10.3389/fimmu.2018.03111

**Published:** 2019-01-22

**Authors:** Jessica Hoppstädter, Britta Diesel, Rebecca Linnenberger, Nina Hachenthal, Sara Flamini, Marie Minet, Petra Leidinger, Christina Backes, Friedrich Grässer, Eckart Meese, Stefano Bruscoli, Carlo Riccardi, Hanno Huwer, Alexandra K. Kiemer

**Affiliations:** ^1^Pharmaceutical Biology, Department of Pharmacy, Saarland University, Saarbrücken, Germany; ^2^Pharmacology, Department of Medicine, Perugia University, Perugia, Italy; ^3^Human Genetics, Department of Medicine, Saarland University, Homburg, Germany; ^4^Chair for Clinical Bioinformatics, Saarland University, Saarbrücken, Germany; ^5^Virology, Department of Medicine, Saarland University, Homburg, Germany; ^6^Cardiothoracic Surgery, Völklingen Heart Centre, Völklingen, Germany

**Keywords:** inflammation, MyD88, TRIF, NF-κB, cytokine, nitric oxide, phagocytosis, microRNA

## Abstract

Activation of toll-like receptors (TLRs) plays a pivotal role in the host defense against bacteria and results in the activation of NF-κB-mediated transcription of proinflammatory mediators. Glucocorticoid-induced leucine zipper (GILZ) is an anti-inflammatory mediator, which inhibits NF-κB activity in macrophages. Thus, we aimed to investigate the regulation and role of GILZ expression in primary human and murine macrophages upon TLR activation. Treatment with TLR agonists, e.g., Pam_3_CSK_4_ (TLR1/2) or LPS (TLR4) rapidly decreased GILZ mRNA and protein levels. In consequence, GILZ downregulation led to enhanced induction of pro-inflammatory mediators, increased phagocytic activity, and a higher capacity to kill intracellular bacteria (*Salmonella* enterica serovar *typhimurium*), as shown in GILZ knockout macrophages. Treatment with the TLR3 ligand polyinosinic: polycytidylic acid [Poly(I:C)] did not affect *GILZ* mRNA levels, although GILZ protein expression was decreased. This effect was paralleled by sensitization toward TLR1/2- and TLR4-agonists. A bioinformatics approach implicated more than 250 miRNAs as potential GILZ regulators. Microarray analysis revealed that the expression of several potentially GILZ-targeting miRNAs was increased after Poly(I:C) treatment in primary human macrophages. We tested the ability of 11 of these miRNAs to target GILZ by luciferase reporter gene assays. Within this small set, four miRNAs (hsa-miR-34b^*^,−222,−320d,−484) were confirmed as GILZ regulators, suggesting that GILZ downregulation upon TLR3 activation is a consequence of the synergistic actions of multiple miRNAs. In summary, our data show that GILZ downregulation promotes macrophage activation. GILZ downregulation occurs both via MyD88-dependent and -independent mechanisms and can involve decreased mRNA or protein stability and an attenuated translation.

## Introduction

Macrophages are strategically distributed all over the body and represent the first line of defense to invading pathogens. In response to bacterial or viral infections, these phagocytic cells engulf and destroy the infectious agent. Macrophages recognize a variety of microbial products, such as bacterial cell wall components and nucleic acids, via pathogen recognition receptors (PRRs). These receptors include Toll-like receptors (TLRs), as well as cytosolic NOD-like receptors, RIG-I-like receptors, and DNA sensors (1, 2).

Once a pathogen is recognized and phagocytosed, macrophages generate a wide range of biologically active molecules, e.g., pro-inflammatory cytokines, chemokines, and growth factors. Cytokines released by macrophages, such as interferons (IFNs), tumor necrosis factor (TNF)-α, and interleukin (IL)-1α/β, stimulate the activity of both the innate and adaptive immune response. Pathogen uptake usually also results in antimicrobial responses such as reactive oxygen species (ROS) and nitric oxide (NO) production, thus enabling a rapid response to infection (3, 4).

Activated macrophages can be deactivated by various mediators, including IL-10, prostaglandins, G-protein coupled receptor ligands, or glucocorticoids (GCs) (3, 4). Anti-inflammatory factors such as IL-10 are also released by macrophages themselves, and failure to produce these mediators can lead to non-resolving inflammation (3, 4).

The Glucocorticoid-induced leucine zipper (GILZ) is an endogenous inhibitor of immune responses. GILZ exerts its anti-inflammatory activity mainly by inhibition of the pro-inflammatory transcription factors nuclear factor (NF)-κB and activator protein (AP)-1 (5–8). In addition, GILZ has been implicated in the negative regulation of mitogen-activated protein kinase (MAPK) signaling (9–11).

Early studies on GILZ focused on its effects on thymocytes and T lymphocytes because of its strong induction by GCs in the thymus (12). Several reports indicated that the pro-apoptotic effects of GILZ overexpression in T lymphocytes mimic GC treatment (5, 8, 12–14). Moreover, GILZ depletion attenuates GC-induced apoptosis in B lymphocytes (15, 16).

In monocytes and macrophages, GILZ can be upregulated by various anti-inflammatory factors, such as GCs, IL-4 or IL-10, and the natural compound curcumin (6, 17–20). In accordance with the GC-like actions of GILZ in lymphocytes, GILZ overexpression in macrophage-like THP-1 cells results in decreased expression of chemokines and macrophage activation markers, as well as NF-κB activity upon treatment with the TLR4 ligand lipopolysaccharide (LPS) (6).

We previously reported that primary human *in vitro* differentiated and *ex vivo* pulmonary macrophages express high constitutive levels of GILZ (11, 21). Both siRNA-mediated GILZ knockdown in human macrophages and GILZ knockout in murine bone marrow-derived macrophages (BMMs) increased the responsiveness toward LPS, suggesting that repression of endogenous GILZ expression represents a positive feedback loop in macrophage activation.

Little is known about the role and regulation of GILZ after stimulation with other TLR ligands, e.g., activators of TLR1/2 or TLR3. The extracellular TLR1/2 heterodimer recognizes bacterial triacetylated lipopeptides and their mimic, the synthetic compound Pam_3_CSK_4_. In contrast, intracellular TLR3 detects double-stranded RNA, i.e., an intermediate in viral replication, as well as its synthetic analogon polyinosinic:polycytidylic acid [Poly(I:C)]. TLRs differentially activate transcription factors due to the varying involvement of the adapter molecules MyD88 (myeloid differentiation primary response gene 88) and TRIF (TIR domain-containing adapter inducing IFN-β). All TLRs except TLR3 can initiate MyD88-dependent signaling, and MyD88-independent signaling via TLR3 or TLR4 utilizes TRIF for signal transduction (1, 2).

In the present study, we provide evidence for dual regulation of GILZ upon MyD88- and TRIF-dependent TLR activation and link GILZ expression levels with pivotal macrophage defense mechanisms, such as phagocytosis and bactericidal activity.

## Materials and Methods

### Materials

Cell media (RPMI1640, #R0883; DMEM, #D6546), fetal calf serum (FCS, #F7524), penicillin/streptomycin (#P433), and glutamine (#G7513) were from Sigma-Aldrich. Anti-GILZ antibodies were obtained from either Santa Cruz Biotechnology (polyclonal goat anti-GILZ Ab, #sc-26518) or eBioscience (CFMKG15, #14-4033-82). The anti-tubulin antibody (#T9026) was obtained from Sigma-Aldrich. Anti-rabbit IRDye 680- and anti-mouse IRDye 800-conjugated secondary antibodies were from LI-COR Biosciences (#926-68071, #926-32210). The anti-rabbit IRDye 800-conjugated secondary antibody was from Rockland (#612-132-120). Anti-p44/42 (ERK1/2) mouse antibody (L34F12, #4696S) and anti-phospho-p44/42 MAPK (Thr202/Tyr204) rabbit mAbs (20G11, #4376S) were obtained from Cell Signaling Technology. TLR ligands, i.e., ultrapure LPS from *Escherichia coli* K12 (#tlrl-peklps), Pam_3_CSK_4_ (#tlrl-pms), lipoteichoic acid (LTA, #tlrl-pslta), and Poly(I:C) (#tlrl-pic) were purchased from Invivogen. Phorbol 12-myristate 13-acetate (PMA, #524400) and BAY 11-7082 (Cay10010266-10) were from Cayman Chemical. BAY 11-7085 (#196872) was obtained from Calbiochem. Human M-CSF (#M6518), MTT (# M5655), actinomycin D (#A9415), and aurintricarboxylic acid (ATA, #A1895) were obtained from Sigma-Aldrich. Murine GM-CSF (#130-095-735), M-CSF (#130-101-704), IFN-γ (#130-105-782), IL-4 (#130-097-761), and TNF-α (#130–101–689) were obtained from Miltenyi Biotec. Primers and dual-labeled probes were from Eurofins MWG Operon. Taq polymerase (5 U/μL, #E00007), Taq buffer (#B0005) and the dNTP mix (#D0056) were from Genscript. D-luciferin was obtained from Applichem (#A1029,0050). Coelenterazine was from Biotium (#10110). Restriction enzymes (BamH1, #R3136S; EcoR1, #R0101S; Sac1, #R0156S; Spe1, #R0133L) were purchased from New England Biolabs. Other chemicals were obtained from either Sigma-Aldrich or Carl Roth unless stated otherwise.

### Mice

Mice were housed in a 12:12 h light-dark cycle with food and water *ad libitum*. Mice expressing Cre recombinase under the control of endogenous *Lyz2* (lysozyme 2) promoter/enhancer elements (LysMcre mice, The Jackson Laboratory, #B6.129P2-Lyz2^tm1(cre)Ifo/J^) were crossed with C57BL/6J mice bearing LoxP sites upstream and downstream of *Gilz* exon 6 (11, 22) to obtain myeloid-specific GILZ KO mice. Genotyping was performed according to protocols provided by The Jackson Laboratory and as described by Bruscoli et al. (22).

### Cell Culture

#### Cell Lines

THP-1 (#TIB202), U937 (#CRL-1593.2), and L929 cells (#CRL-6364) were obtained from ATCC and grown in standard medium (RPMI 1640, 10% FCS, 100 U/mL penicillin G, 100 μg/mL streptomycin, 2 mM glutamine). THP-1 and U937 were differentiated into macrophage-like cells by treatment with PMA (100 nM) for 48 h. HEK293T cells (ATCC, #CRL-3216) were cultured in high glucose DMEM medium with supplements (10% FCS, 100 U/mL penicillin G, 100 μg/mL streptomycin, 2 mM glutamine). HEK-Blue™ Null1 cells (Invivogen, #hkb-null1) were grown in high glucose DMEM medium supplemented with 10% FCS, 2 mM glutamine, 50 U/mL penicillin G, 50 μg/mL streptomycin, 100 μg/mL Normocin (Invivogen, #ant-nr-1), and 100 μg/mL Zeocin (Invivogen, #ant-zn-1).

#### Human Alveolar Macrophages (AMs)

AMs were isolated from human non-tumor lung tissue obtained from patients undergoing lung resection. The use of human material for isolation of primary cells was reviewed and approved by the local Ethics Committees (permission no. 213/06; State Medical Board of Registration, Saarland, Germany). The informed consent of all participating subjects was obtained. Isolation was performed according to a previously described method (23) with minor modifications. After visible bronchi were removed, the lung tissue was cut into pieces (~1 cm^3^) and washed at least three times with BSS (balanced salt solution; 137 mM NaCl, 5 mM KCl, 0.7 mM Na_2_HPO_4_, 10 mM HEPES, 5.5 mM glucose, pH 7.4). The washing buffer was collected and centrifuged (15 min, 350 x *g*). Erythrocytes were lysed by incubation with hypotonic buffer (155 mM NH_4_Cl, 10 mM KHCO_3_, 1 mM Na_2_EDTA) for 2–5 min at 37°C, and the cell suspension was washed with PBS (137 mM NaCl, 2.7 mM KCl, 10.1 mM Na_2_HPO_4_, 1.8 mM KH_2_PO_4_, pH 7.4). Cells were resuspended in AM medium (RPMI 1640, 5% FCS, 100 U/mL penicillin G, 100 μg/mL streptomycin, 2 mM glutamine), seeded at a density of 0.5–1 × 10^6^ cells/well into a 12- or 6-well plate and incubated at 37°C for 2 h. Adherent cells were washed at least 5 times with PBS, and cells were cultured in AM medium overnight before further use. AM preparations were 95% pure as assessed by flow-cytometric analysis of intracellular CD68 expression (23, 24).

#### Human Monocyte-Derived Macrophages

Monocytes were isolated from healthy adult blood donors (Blood Donation Center, Saarbrücken, Germany) and differentiated with M-CSF as described in Dembek et al. (25). The use of the human material was reviewed and approved by the local Ethics Committees (permission no. 130/08; State Medical Board of Registration, Saarland, Germany). Peripheral blood mononuclear cells (PBMCs) were isolated by density gradient centrifugation using Lymphocyte Separation Medium 1077 (Promocell, #C-44010) and Leucosep tubes (Greiner Bio-One, #227290) as recommended by the suppliers. After washing with PBS, monocytes were purified from PBMCs by magnetic cell sorting using anti-CD14 microbeads (Miltenyi, #130-050-201) according to the manufacturer's instructions, except that 10% of the recommended bead amount was used (25). Monocyte purity was > 95% as indicated by CD14 expression (data not shown). Monocytes were seeded into 12 well plates (5 × 10^5^ cells/well) and differentiated into macrophages in RPMI 1640 supplemented with 10% FCS, 100 U/mL penicillin G, 100 mg/mL streptomycin, 2 mM glutamine, and 20 ng/mL M-CSF at 37°C and 5% CO_2_ for 7 d. The medium was changed every other day.

#### Murine Bone Marrow-Derived Macrophages (BMMs)

BMMs were obtained from 8 to 12-week-old male wild-type (WT, LysMCre^+/+^/floxed GILZ^−/y^) or GILZ knockout (KO, LysMCre^+/+^/floxed GILZ^+/y^) mice as described previously with minor modifications (11). Femurs and tibias were flushed with standard medium (RPMI 1640, 10% FCS, 100 U/mL penicillin G, 100 μg/mL streptomycin, 2 mM glutamine). After centrifugation (10 min, 250 x *g*), erythrocytes were lysed by incubation in hypotonic buffer (155 mM NH_4_Cl, 10 mM KHCO_3_, 1 mM Na_2_EDTA) for 2 min at 37°C. Cells were washed with PBS, resuspended in standard medium containing M-CSF (50 ng/mL, 30 mL per preparation), transferred into a 75 cm^2^ culture flask and allowed to adhere overnight. Non-adherent cells were collected and cultured in a 150 cm^2^ culture flask for another 5 d in M-CSF-containing medium. Differentiated cells were detached with accutase (Sigma-Aldrich, #A6964), suspended in standard medium supplemented with 10 ng/mL M-CSF, and seeded into 96-well plates (7.5 × 10^4^ cells/well) for TNF measurements or into 12-well plates (5 × 10^5^ cells per well) for all other applications unless indicated otherwise. BMMs were > 95% pure as indicated by flow cytometric analysis using an antibody against F4/80 (data not shown).

#### MPI Cells

MPI cells, i.e., non-transformed self-renewing primary murine macrophages, were obtained from WT or GILZ KO mice based on a previously described method (26). MPI cells were prepared from fetal livers of 15-d-old mouse embryos by pushing the tissue through 70 μm cell strainers while washing with PBS. Cells were washed once with PBS and then resuspended in standard medium (RPMI 1640, 10% FCS, 100 U/mL penicillin G, 100 μg/mL streptomycin, 2 mM glutamine) supplemented with 30 ng/mL GM-CSF. Proliferating cells were subcultured by splitting them 1:5 after 6–8 d. Cell preparations were > 95% positive for macrophage markers F4/80 and CD68, as indicated by flow cytometric analysis (data not shown). Cells were seeded into 96-well plates (4 × 10^4^ cells/well) for determination of NO release or into 12 well plates (2 × 10^5^ cells/well) for phagocytosis assays.

### Determination of Cell Viability

The MTT colorimetric assay was performed to ensure that non-toxic concentrations of U0126, BAY-11-7082, BAY-11-7085, and ATA were used. Cells were incubated for 24 h with the test compounds diluted in a cell-type specific medium. After that, cells were incubated with MTT solution (5 mg/mL in medium) for 2 h. The supernatant was discarded, and cells were lysed in 100 μL DMSO. Absorbance measurements were carried out at 550 nm with 630 nm as the reference wavelength using a microplate reader (Tecan Sunrise). The cell viability obtained from at least two independent experiments was calculated relative to untreated and solvent controls (data not shown).

### Cytokine Quantification

Cytokines were quantified in AM supernatants using a Milliplex MAP Human Cytokine Kit (Millipore, #HCYTOMAG-60K) as detailed in Hoppstädter et al. (23). AMs were kept at a density of 1 × 10^5^ cells per well in 96 well plates in a total volume of 100 μL medium in the presence or absence of Pam_3_CSK_4_ (100 ng/mL) or Poly(I:C) (10 μg/mL) for 6 h. The supernatants were collected and stored at −80°C until they were used in the multiplex cytokine assay. The immunoassay procedure was performed using a serial dilution of the 10,000 pg/mL human cytokine standard according to the manufacturer's instructions, and the plate was read at the Luminex 200 System (Millipore).

Murine TNF-α was quantified by bioassay as previously described (11). L929 cells were seeded at a density of 3 × 10^4^ cells per well into a 96-well plate. After 24 h, the medium was replaced by 100 μL of actinomycin D solution (1 μg/mL in standard medium) and cells were incubated for 1 h at 37°C. Subsequently, BMM supernatants (100 μL per well) were added. Dilution series of recombinant murine TNF-α (100–2,500 pg/mL) were run alongside the samples to generate a standard curve. The plates were incubated for an additional 24 h at 37°C. The MTT assay (see Determination of Cell Viability) was used to assess cell viability.

### RNA Isolation, Reverse Transcription, and Quantitative RT-PCR

Total RNA was isolated using the RNeasy Plus Mini Kit (Qiagen, #74134), and 200 ng RNA were reverse transcribed using the High-Capacity cDNA Reverse Transcription Kit (Applied Biosystems, #4368813) according to the manufacturer's recommendations. The cDNA was diluted with 80 μL TE buffer (Applichem, #A0386) before use. The CFX96 Touch™ Real-Time PCR Detection System (Bio-Rad) was used for real-time RT-PCR. For *TNF, IL6, CXCL8, GILZ*, and *ACTB*, one 25 μL reaction mix contained 2.5 U Taq polymerase, 500 nM sense and antisense primers, 60–100 nM probe, 200 μM dNTPs, 3–4 mM MgCl_2_, 2.5 μL 10x Taq buffer, 3 μL Template, and molecular biology grade water (Applichem, #A7398). The reaction conditions were 95°C for 8 min followed by 40 cycles of 15 s at 95°C, 15 s at a reaction dependent temperature varying from 57 to 60°C, and 15 s at 72°C. For *ZFP36* detection, the 5x HOT FIREPol® EvaGreen® qPCR Mix Plus (Solis Biodyne, #08-25) was used according to the manufacturer's recommendations. Primer and probe sequences as well as specific reaction conditions are given in Supplementary Table 1. Standard curves were generated by serial dilution of the PCR product cloned into pGEMTeasy (Promega, #A1360). All samples and standards were analyzed in triplicate.

### Western Blotting

Cells were lysed in lysis buffer [50 mM Tris-HCl, 1% (m/v) SDS, 10% (v/v) glycerol, 5% (v/v) 2-mercaptoethanol, 0.004% (m/v) bromphenol blue] supplemented with a protease inhibitor mix (cOmplete; Roche Diagnostics, #04693124001) and stored at −80°C until further use. After sonication, lysates were boiled for 5 min at 95°C. Proteins were separated by SDS-PAGE on 12–15% gels using the Mini-Protean Tetra Cell system (BioRad) and transferred onto Immobilon FL-PVDF membranes (Millipore, #IPFL00010) using the Tetra Blotting Module (BioRad). The membranes were blocked in blocking buffer for near-infrared Western Blotting (Rockland, MB070) for 1 h, incubated with primary antibody dilutions (1:500–1:2,000 in Rockland blocking buffer) for 3 h at room temperature or at 4°C overnight and with IRDye 680 or IRDye 800 conjugated secondary antibodies (1:5,000–1:10,000) for 1.5 h at room temperature. Blots were scanned with an Odyssey Infrared Imaging System (LI-COR Bioscience), and relative protein amounts were determined using either Odyssey or ImageJ software.

### Griess Assay

MPI cells were cultured in 96-well plates (4 × 10^4^ cells per well) and treated as indicated. After 20 h, the concentration of nitrite was measured in the supernatants by Griess assay as previously described (20). In brief, 90 μL 1% sulfanilamide in 5% H_3_PO_4_ and 90 μL 0.1% N-(1-naphthyl)ethylenediamine dihydrochloride in H_2_O were added to 100 μL of the cell culture supernatant, followed by absorbance measurement at 550 nm and the reference wavelength 630 nm using a Tecan Sunrise microplate reader. A standard curve of sodium nitrite dissolved in the medium was run alongside the samples. Total cellular protein concentrations used for data normalization were determined by Pierce BCA protein assay (ThermoFisher Scientific, #23225) according to the manufacturer's instructions.

### Phagocytosis Assay

Particle uptake was quantified after incubation of macrophages with 1.75 μm latex beads (Fluoresbrite Carboxylated YG microspheres; Polysciences, #17687) at a 50:1 bead/cell ratio in full medium. Subsequently, cells were washed 5 times with ice-cold PBS to remove remaining fluorospheres and detached from plates using ice-cold TEN buffer (40 mM Tris, 1 mM EDTA, 150 mM NaCl). Cells were resuspended in ice-cold PBS (137 mM NaCl, 2.7 mM KCl, 10.1 mM Na_2_HPO_4_, 1.8 mM KH_2_PO_4_, pH 7.4) and particle-associated fluorescence was determined using an LSR Fortessa flow cytometer (BD Biosciences). Particle uptake was verified by quenching the fluorescence of particles that were merely attached with trypan blue solution (1 mg/mL in PBS, data not shown).

### *Salmonella* Infection and Determination of Colony Forming Units (CFU)

The *Salmonella* enterica serovar *typhimurium* (*S. typhimurium*) wild-type strain 12023 (resp. 14028s) was a gift from D. Monack (Stanford University). *S. typhimurium* was grown to stationary phase overnight in LB medium (Carl Roth, #X968.1) at 37°C with aeration. 2 × 10^5^ BMMs per well were seeded into a 24-well plate, followed by infection at an MOI of 100:1. 30 min post-infection, gentamycin (100 μg/mL, Sigma-Aldrich, #G1397) was added to kill extracellular bacteria. One hundred and twenty minutes post-infection, fresh medium containing 10 μg/mL gentamycin was added to the cells. Eight hours post-infection, cells were lysed in 200 μL 1% Triton X-100 (Sigma-Aldrich, #T8787) in water and 1:10 serial dilutions of bacteria suspensions were plated on LB plates. Colony-forming units (CFU) were determined the following day in 1:10 and 1:100 dilutions and the dilution factor was included in CFU calculations.

### miRNA Detection

Total RNA for miRNA detection was isolated from AMs using the miRNEasy Kit (Qiagen, #217004). The RNA quality was assessed using the Agilent Bioanalyzer 2100 and RNA 6000 Pico Chips (Agilent Technologies, #5067-1513) as recommended by the supplier. For miRNA expression analysis, the Agilent microarray platform and Human miRNA 8x60K microarrays (Release 16.0, Agilent Technologies, #G4870A) were used according to manufacturer's instructions. In brief, 100 ng total RNA and the miRNA Complete Labeling and Hyb Kit (Agilent Technologies, #5190-0456) were used to generate fluorescently labeled miRNA. The microarrays were loaded and incubated for 20 h at 55°C and 20 rpm. After several washing steps, the microarrays were scanned with the Agilent Microarray Scanner at 3 microns in double path mode. The Total Gene Signal provided by the Agilent Feature Extraction software was used for quantitative data analysis. Normalization between arrays was carried out using quantile normalization. The data have been deposited in NCBI's Gene Expression Omnibus (27) and are accessible through GEO Series accession number GSE123756.

### Luciferase Reporter Gene Assay

miRNA overexpression vectors were generated by cloning the miRNA precursor sequences into the BamH1/EcoR1 sites of pSG5 (Agilent Technologies, #216201) using the primers given in Supplementary Table 2 or as previously described (28–30). GILZ 3′UTR-luciferase reporter constructs were generated by coupling the GILZ 3′UTR sequence to a firefly luciferase reporter gene (21). Human GILZ 3′UTR cDNA was amplified using the Expand High fidelity PCR System (Sigma-Aldrich, # 11732641001) and the following primers: 5′-GCC TAC TAG TGC AGA GCC ACT AAA CTT G-3′ and 5′-AAT AGA GCT CAC TCT CAC AAA ACC CGC TAC-3′. The SacI/SpeI digested PCR product was inserted into the respective cloning site of pMIR-REPORT (Ambion, #AM5795). Plasmid DNA was purified from overnight cultures with the Genopure Plasmid Midi Kit (Roche, #3143414001). Reporter gene assays were performed by transfecting HEK293T cells with the pMIR-REPORT reporter construct, a pre-miRNA vector, and phRG-TK Renilla (Promega, #E6291). HEK 293T cells were seeded at a density of 2 × 10^4^ cells per well into a 96-well-plate, grown for 24 h and transfected using PolyFect (Qiagen, #301105) according to the manufacturer's recommendations. Twenty-four hours after transfection, cells were lysed with 5x Passive Lysis Buffer (Promega, #E1941). Luciferase activity was determined after the addition of firefly luciferase substrate (470 μM D-luciferin, 530 μM ATP, 270 μM coenzyme A, 33 mM DTT, 20 mM Tricine, 2.67 mM MgSO_4_, and 0.1 mM EDTA, pH 7.8) or renilla substrate (0.1 M NaCl, 25 mM Tris-HCl, pH 7.5, 1 mM CaCl_2_, 1 μM coelenterazine) by luminescence measurement using a Glomax Discover multiplate reader (Promega). Firefly luciferase activity was normalized to renilla luciferase activity to correct for variations in transfection efficiency, and relative luminescence was calculated relative to PSG5 control transfected cells. Data were also normalized to the impact of each miRNA on the luciferase mRNA without GILZ 3′UTR to adjust for GILZ 3′UTR-specific effects.

### miRNA Mimic Transfection

miRNA mimics (hsa-miR-34b^*^,−222,−320d,−484, and scrambled control; MISSION miRNA, Sigma-Aldrich, #HMI0510, #HMI0400, #HMI0475, #HMI0593, #HMC0002) were reverse transfected into HEK293T cells (2.5 × 10^5^ cells in a 6-well plate) using the Lipofectamine RNAiMAX transfection reagent (ThermoFisher Scientific, #13778075) as recommended by the supplier. Mimics were used at a final concentration of 50 nM. To examine potential synergistic effects, a combination of all four miRNA mimics (12.5 nM each) was compared with scrambled controls (50 nM) or miR-34b^*^ mimic (12.5 nM) only. The latter was co-transfected with scrambled control mimics (37.5 nM) to ensure comparable transfection conditions.

### NF-κB/AP-1 Reporter Gene Assay

HEK-Blue cells (HEK-Blue Blue™ Null1, Invivogen, #hkb-null1) expressing secreted embryonic alkaline phosphatase (SEAP) under the control of the IFN-β minimal promoter fused to five NF-κB and AP-1 binding sites were used to determine NF-κB/AP-1 activity. Cells were seeded into 96-well plates (5 × 10^5^ cells/well) and treated immediately as indicated. After 24 h, supernatants were collected, and SEAP activity was determined using the QuantiBlue reagent (Invivogen, #REP-QB2) according to the supplier's instructions. For miRNA mimic experiments, HEK-Blue cells (2 × 10^5^ cells/well in 96-well plates) were reverse transfected with single miRNA mimics (50 nM), scrambled control (50 nM), or a mix of miR-34b^*^,−222,−320d, and−484 mimics (12.5 nM, each) using the Lipofectamine RNAiMAX transfection reagent as recommended by the supplier (see miRNA Mimic Transfection for details). Twenty-four hours later, cells were treated as indicated and incubated for another 24 h. Subsequently, SEAP activity was determined as described above.

### TAT-Fusion Proteins

The cell-permeable trans-activator of transcription peptide (TAT)-GILZ fusion protein and the respective control protein (TAT-Co) were generated and purified as previously (13, 31). In brief, TAT and TAT-GILZ sequences were inserted into the pGEX-4T2 plasmid (GE Healthcare, #28-9545-50) to produce an in-frame fusion protein. The GST fusion protein expression was induced in *E. coli* BL21 (GE Healthcare, #27-1542-01) with 0.1 mM isopropyl β-d-thiogalactopyranoside (Sigma-Aldrich, #I5502). After lysis by sonication, proteins were purified with glutathione-sepharose 4B beads (GE Healthcare, #17-0756-01) according to the manufacturer's instructions. Protein purity was evaluated by SDS-PAGE and Coomassie blue staining.

### Statistics

All experiments were performed at least three times, and at least two biological replicates were analyzed for all *in vitro* experiments unless stated otherwise. Data distribution was determined by the Shapiro-Wilk test. For normally distributed data, means of two groups were compared with non-paired two-tailed Student's *t*-test. For data that were not normally distributed, means of two groups were compared using the Mann-Whitney test. Means of more than two groups were compared by one-way ANOVA with Bonferroni's *post hoc* test (normal distribution) or Kruskal-Wallis ANOVA followed by Mann-Whitney test (no normal distribution). Statistical significance was set at *p* < 0.05, *p* < 0.01, or *p* < 0.001. Data analysis was performed using Origin software (OriginPro 8.6G; OriginLab).

## Results

### Downregulation of GILZ by TLR Agonists

**Figure 1:**
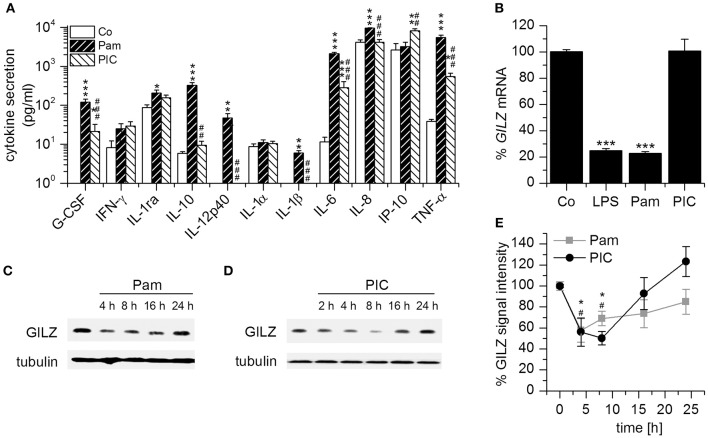
GILZ is downregulated in AMs upon MyD88-dependent and MyD88-independent TLR activation. **(A)** Responsiveness of AMs toward the TLR1/2 ligand Pam_3_CSK_4_ (Pam, 100 ng/mL, 6 h) and the TLR3 ligand Poly(I:C) (PIC, 10 μg/mL, 6 h). Cytokine production was determined by Luminex bead assay (*n* = 4, triplicates; *p* < 0.05, ^**^p < 0.01, ^***^p < 0.001 compared with Co; ^#^*p* < 0.05, ^##^*p* < 0.01, ^###^*p* < 0.001 compared with Pam-treated AMs). **(B)** AMs were left untreated or treated with LPS (1 μg/mL), Pam (1 μg/mL) or PIC (10 μg/mL) for 2 h and *GILZ* mRNA was quantified by qRT-PCR using *ACTB* as a housekeeping gene (*n* = 4, duplicates; ^***^p < 0.001 compared with Co). **(C–E)**: AMs were treated with Pam (**C,E**; 1 μg/mL) or PIC (**D,E**; 10 μg/mL) for the indicated time points and GILZ levels were determined by Western blot. Tubulin served as a loading control. **(C,D)** Representative blots. **(E)** GILZ signal intensities were quantified and normalized to tubulin values [*n* = 3; ^*^,^#^*p* < 0.05 compared with 0 h for Pam (^*^) and PIC (^#^)-treated cells].

Because most studies on GILZ focused on its pharmacological induction, there are only limited data on the regulation of endogenous GILZ during the immune response. To investigate macrophage responses toward TLR1/2 and TLR3 ligands, we treated human AMs, human monocyte-derived macrophages, as well as non-differentiated and PMA-differentiated U937 and THP-1 cells with Pam_3_CSK_4_ and Poly(I:C). Out of these human macrophages and macrophage-like cells, AMs were the only cell type that showed a robust activation by Poly(I:C) regarding cytokine production Figure 1A, Supplementary Figure 1). Thus, we used these cells to further examine GILZ expression. As reported previously (21), *GILZ* mRNA levels were highly reduced upon TLR1/2 and TLR4, but not TLR3 stimulation (Figure 1B). In contrast, GILZ protein levels decreased both after MyD88-dependent stimulation by Pam_3_CSK_4_ and TRIF-dependent activation by Poly(I:C) (Figures 1C–E), implicating a dual regulation of GILZ.

**Figure 2:**
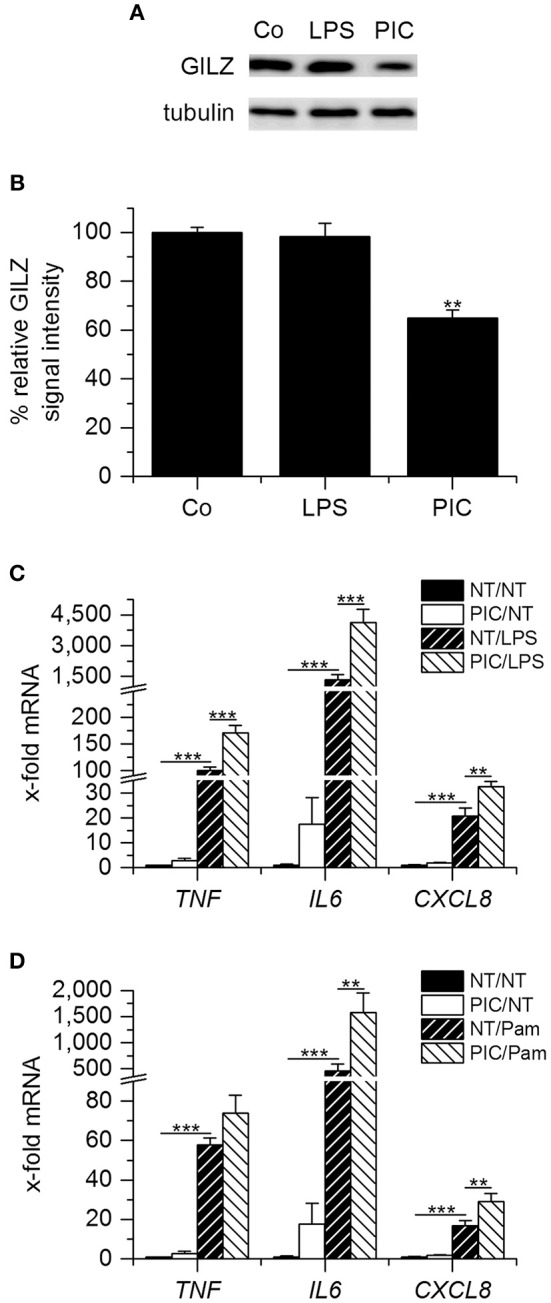
Pretreatment with low-dose Poly(I:C) reduces GILZ expression paralleled by sensitization toward LPS and Pam_3_CSK_4_. **(A,B)** AMs were left untreated (Co) or treated with LPS (100 ng/mL) or Poly(I:C) (PIC, 1 μg/mL) for 24 h. GILZ levels were determined by Western blot using tubulin as a loading control. **(A)** Representative blot. **(B)** GILZ signal intensities were quantified and normalized to tubulin values (*n* = 3, duplicates; ^**^p < 0.01 compared with Co). **(C,D)** AMs were either left untreated (NT) or pretreated with PIC (1 μg/mL), followed by stimulation with LPS (1 μg/mL, **C**) or Pam_3_CSK_4_ (Pam, 1 μg/mL, **D**) for another 2 h. *TNF, IL6*, and *CXCL8* mRNA expression was determined by qRT-PCR using *ACTB* as a housekeeping gene (*n* = 4, duplicates; ^**^p < 0.01, ^***^p < 0.001).

During the late stages of sepsis, macrophages adjust to the prolonged stimulation by LPS or other TLR ligands and enter a hyporesponsive state termed endotoxin or LPS tolerance. LPS-tolerant macrophages are characterized by reduced secretion of pro-inflammatory cytokines and upregulation of anti-inflammatory genes. We previously showed that GILZ represents a key mediator of LPS tolerance (11). In contrast, pretreatment of AMs with low-dose Poly(I:C) for 24 h resulted in reduced GILZ levels (Figures 2A,B) and an enhanced LPS- or Pam_3_CSK_4_-induced pro-inflammatory response, as shown by qPCR analysis of *TNF, IL6*, and *CXCL8* expression (Figures 2C,D). This observation is in line with a recent report showing that macrophages generate a type of innate immune memory by activating the TLR3 pathway, thereby enhancing the immune response to a subsequent PAMP exposure (32). Our data suggest that this might, at least in part, be due to GILZ downregulation.

### Functional Implications of Gilz Downregulation

**Figure 3:**
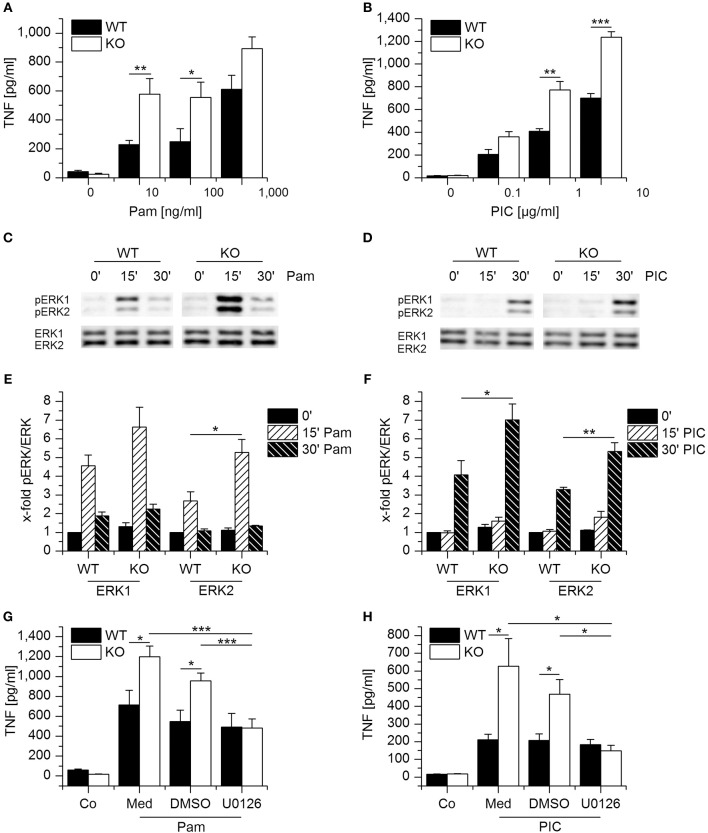
Loss of GILZ increases the responsiveness of macrophages toward Pam_3_CSK_4_ and Poly(I:C). **(A,B)** Wildtype- (WT) and GILZ Knockout-(KO) BMMs were treated with Pam_3_CSK_4_ (4 h, **A**) or Poly(I:C) (6 h, **B**) at the indicated concentrations. TNF secretion was quantified by bioassay (*n* = 4, triplicates). **(C–F)** WT and GILZ KO BMMs were treated with Pam_3_CSK_4_ (100 ng/mL, C and E) or Poly(I:C) (10 μg/mL, **D,F**) for the indicated periods of time. ERK activation was determined by Western blot. **(C,D)** Representative blots. **(E,F)** pERK signal intensities were quantified and normalized to total ERK signals. Values for untreated WT cells (0′) were set as 1 (*n* ≥ 3). **(G, H)**: WT and GILZ KO BMMs were pretreated with medium only (Med), the solvent control DMSO (0.1%), or U0126 (10 μM) for 2 h, followed by activation with Pam_3_CSK_4_ (100 ng/mL, 4 h; G) or Poly(I:C) (10 μg/mL, 6 h). TNF production was assessed by bioassay (Co: untreated cells; *n* = 4, triplicates). ^*^p < 0.05, ^**^p < 0.01, ^***^p < 0.001.

We then sought to characterize the functional implications of GILZ downregulation. To examine whether lack of GILZ modulates macrophage activation after Pam_3_CSK_4_ and Poly(I:C), we treated murine WT and GILZ KO macrophages with increasing dosages of both stimuli, and TNF-α secretion was quantified. Compared with their WT counterparts, GILZ KO cells released higher levels of TNF-α after stimulation with either TLR ligand (Figures 3A,B). Loss of GILZ has been shown to enhance LPS-induced ERK-signaling in macrophages and thereby promote inflammatory actions, including TNF-α production (11). Therefore, we investigated Pam_3_CSK_4_- and Poly(I:C)-mediated ERK phosphorylation. We detected ERK activation after treatment with both TLR ligands, and GILZ KO BMMs showed higher levels of pERK after 15 min for Pam_3_CSK_4_ and after 30 min for Poly(I:C) when compared with equally treated WT cells (Figures 3C–F).

Since ERK activity might play a role in increased cytokine expression concomitant with GILZ KO, we blocked ERK activation with the inhibitor U0126 and quantified TNF-α after Pam_3_CSK_4_ or Poly(I:C) treatment. Inhibition of ERK signaling abolished the increased responsiveness of GILZ KO cells toward both ligands, indicating that loss of GILZ enhances the inflammatory response by modulating ERK activity (Figures 3G,H).

**Figure 4:**
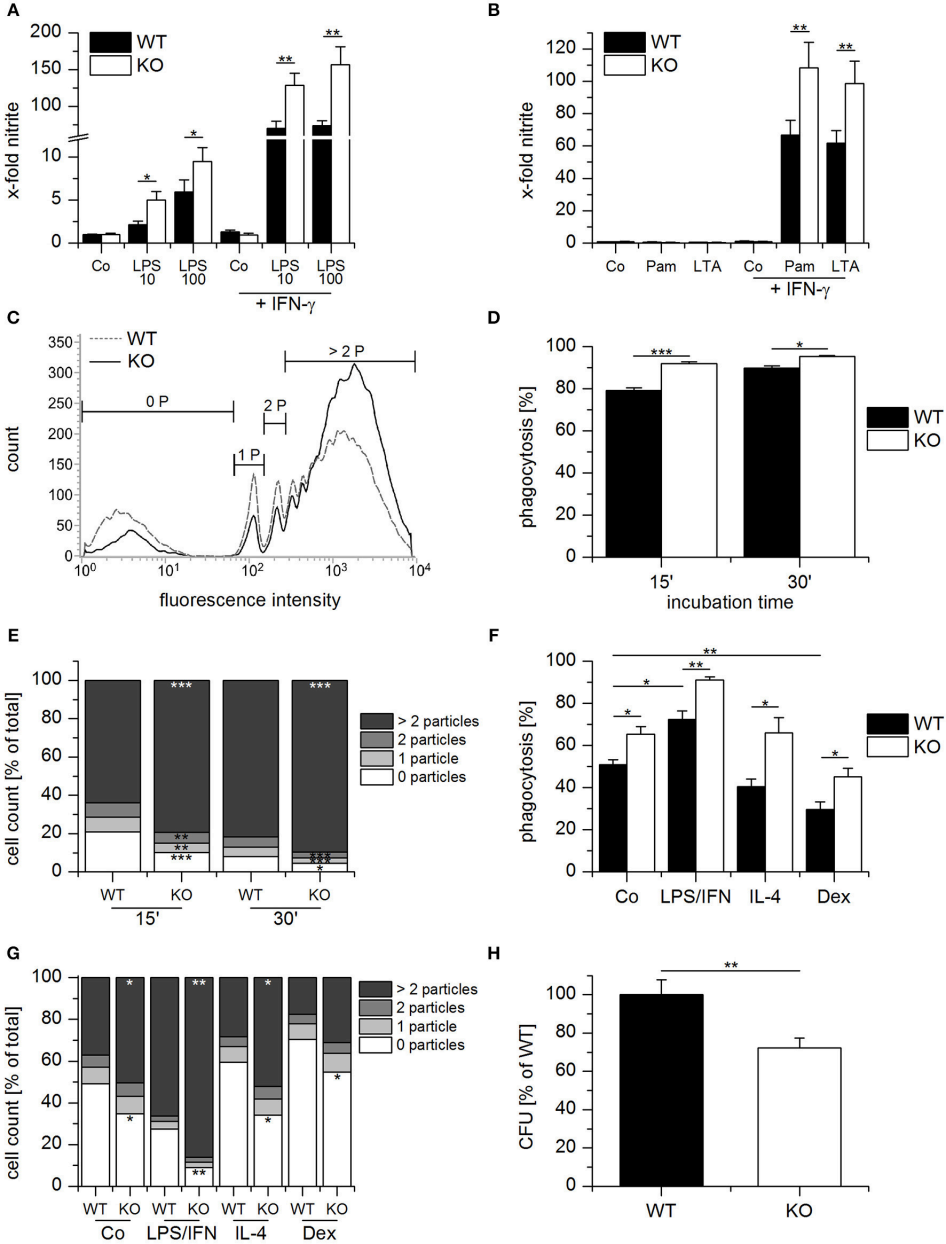
Anti-bacterial host defense is enhanced in GILZ knockout macrophages. **(A,B)** Wildtype- (WT) and GILZ Knockout- (KO) MPI macrophages were treated with LPS (10 or 100 ng/mL) Pam_3_CSK_4_ (Pam, 1 μg/mL, **B**), or lipoteichoic acid (LTA, 5 μg/mL, **B**) with or without co-stimulation by IFN-γ (20 ng/mL) for 20 h. NO-production was measured by Griess assay. Data were normalized to total protein concentration and expressed as x-fold of untreated cells (*n* = 4, triplicates). **(C–E)** Phagocytic activity of WT and GILZ KO MPI cells. MPI cells were incubated with fluorescent latex particles (diameter 1.75 μm, 50 particles per cell) for 15 or 30 min and particle-associated fluorescence was quantified by flow cytometry (15 min: *n* = 2, 30 min: *n* = 6, triplicates). **(C)** Representative histogram for 15 min. 0 P, cells without particles; 1 P, 1 particle; 2 P, 2 particles; > 2 P, more than 2 particles. **(G)** Phagocytic activity as a total percentage of cells with particles. **(E)** The percentage of cells that engulfed 0, 1, 2 ore more than 2 particles was quantified as shown in **(C)**. **(F,G)** WT and GILZ KO BMMs were left untreated (Co) or treated with LPS (1 μg/mL) and INF-γ (IFN, 20 ng/mL), IL-4 (20 ng/mL) or dexamethasone (Dex, 1 μM) for 20 h, followed by incubation with fluorescent latex particles (diameter 1.75 μm, 50 particles per cell) for 1 h. Particle uptake was quantified by flow cytometry. **(F)** Total percentage of cells with particle-associated fluorescence. **(D)** Percentage of cells with 0, 1, 2 or more than 2 particles. **(H)** Relative number of viable bacteria in *Salmonella typhimurium*-infected WT and GILZ KO BMMs. Extracellular bacteria were removed after 30 min, and the remaining bacteria were killed using gentamicin. Eight hours following infection, monolayers were lysed, and the number of intracellular bacteria was determined as Colony Forming Units (CFU) (*n* = 8, triplicates). ^*^p < 0.05, ^**^p < 0.01, ^***^p < 0.001 as indicated or compared with equally treated WT cells **(E,G)**.

Apart from the production of inflammatory cytokines, macrophage-dependent host defense involves the release of nitric oxide (NO) and phagocytosis of the invading pathogen. Thus, we examined the influence of GILZ expression on these parameters. NO production was elevated in GILZ KO cells when compared with equally treated WT cells upon TLR4 activation by LPS alone or in combination with IFN-γ (Figure 4A). TLR2 ligands, i.e., Pam_3_CSK_4_ or lipoteichoic acid (LTA), induced NO only when combined with IFN-γ. As seen for LPS, the response was higher in GILZ KO macrophages (Figure 4B).

To assess their phagocytic capacity, WT and GILZ KO macrophages were incubated with fluorescent latex particles. The total percentage of cells with particle-associated fluorescence and the percentage of cells that engulfed 0, 1, 2, or more than 2 particles was determined by flow cytometry after 15 and 30 min (Figures 4C–E). GILZ KO macrophages phagocytosed more particles than WT cells, which was especially evident in the fraction that took up more than two particles.

To determine the influence of GILZ expression on the phagocytic activity of differentially polarized macrophages, WT, and GILZ KO macrophages were either left untreated or pretreated with LPS/IFN-γ, IL-4, or dexamethasone for 20 h. Subsequently, latex beads were added, and uptake was quantified by flow cytometry. Phagocytosis was enhanced by LPS/IFN-γ and impaired by dexamethasone treatment. Regardless of the treatment scheme, GILZ KO macrophages had a higher uptake efficiency than equally treated WT cells (Figures 4F,G and Supplementary Figure 2).

Next, we investigated whether the loss of GILZ modulated the bactericidal capacity. WT and GILZ KO BMMs were exposed to *Salmonella typhimurium*, and extracellular bacteria were removed after 30 min. After 8 h the number of viable bacteria within the cells was determined. GILZ KO macrophages were more efficient regarding the killing of intracellular bacteria, as indicated by lower CFU counts when compared with WT macrophages (Figure 4H).

### Mechanisms of GILZ Downregulation

**Figure 5:**
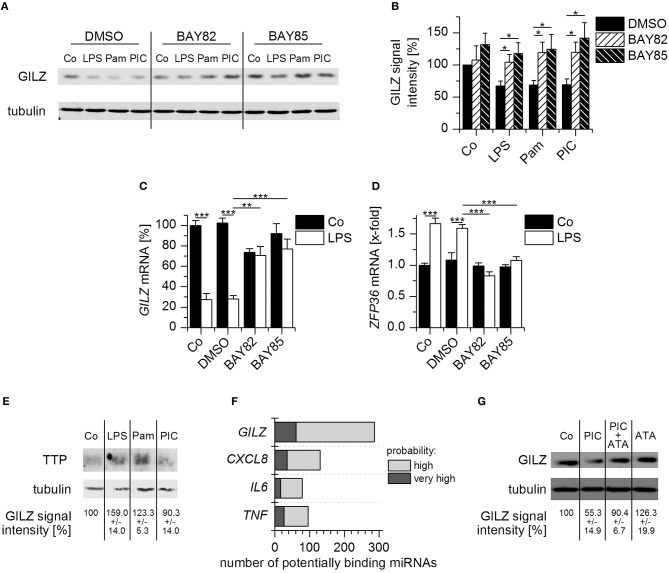
Mechanisms of GILZ downregulation upon TLR activation. **(A,B)** AMs pretreated with BAY-11-7082 (BAY82, 5 μM), BAY-11-7085 (BAY85, 5 μM), or the solvent control DMSO (0.1%) for 1 h, followed by treatment with LPS (100 ng/mL), Pam_3_CSK_4_ (Pam, 1 μg/mL), Poly(I:C) (PIC, 10 μg/mL), or medium (Co) for 4 h. GILZ expression was determined by Western blot. Tubulin served as a loading control. **(A)** Representative blot. **(B)** GILZ signal intensities were quantified and normalized to tubulin values (*n* = 7). Values for unstimulated DMSO controls were set as 100%. **(C,D)** After preincubation with BAY-11-7082 or BAY-11-7085 (5 μM, 1 h), solvent (0.1% DMSO) or medium only (Co), AMs were treated with LPS (100 ng/mL) for 2 h. *GILZ* and *ZFP36* mRNA expression was determined by qRT-PCR using *ACTB* as a housekeeping gene (*n* = 3, duplicates). **(E)** AMs were either left untreated (Co) or treated with LPS (100 ng/mL), Pam_3_CSK_4_ (Pam, 1 μg/mL), or Poly(I:C) (PIC, 10 μg/mL) for 4 h. TTP levels were determined by Western blot using tubulin as a loading control. GILZ signal intensities were normalized to tubulin and are shown as a percentage of untreated cells (*n* = 2, triplicates). **(F)** The number of miRNAs predicted to target *GILZ, CXCL8, IL6*, and *TNF* was assessed via the microRNA Data Integration Portal (mirDIP, accession date 02/02/2018). **(G)** AMs were either left untreated (Co) or treated with Poly(I:C) (PIC, 10 μg/mL), aurintricarboxylic acid (ATA, 25 μM), or a combination of both for 8 h. GILZ expression was quantified by Western blot. Signal intensities were normalized to tubulin and expressed as a percentage of untreated cells (*n* = 3). ^*^p < 0.05, ^**^p < 0.01, ^***^p < 0.001.

We then aimed to address the mechanisms underlying GILZ downregulation in activated macrophages. Both the MyD88- and TRIF-dependent pathways can lead to the activation of the pro-inflammatory transcription factor NF-κB. Thus, we blocked NF-κB signaling in AMs by pretreatment with the inhibitors BAY-11-7082 or BAY-11-7085, followed by treatment with the TLR ligands LPS, Pam_3_CSK_4_, and Poly(I:C), and found that GILZ downregulation was abrogated in inhibitor-treated cells (Figures 5A,B). The efficiency of the inhibitor treatment was verified by the impairment of LPS-induced *TNF* production (Supplementary Figure 3). As seen previously, LPS treatment also resulted in downregulation of *GILZ* on the mRNA level. The effect was reversible by inhibition of NF-κB (Figure 5C), suggesting an involvement of an NF-κB-inducible factor in *GILZ* mRNA regulation. We previously reported that downregulation of GILZ upon MyD88-dependent TLR activation required the presence of the RNA-binding protein tristetraprolin (TTP, encoded by *ZFP36*) (11, 21). Therefore, we speculated that TTP might be, at least in part, regulated via NF-κB. We found indeed that *ZFP36* mRNA was induced upon LPS treatment in an NF-κB-dependent fashion (Figure 5D).

In line with the finding that Poly(I:C) was unable to reduce *GILZ* mRNA levels, we observed no induction of TTP after Poly(I:C) treatment (Figure 5E), suggesting an entirely different mode of action.

miRNAs are endogenous small noncoding RNAs that can facilitate translational repression (33). A bioinformatics approach implicated more than 250 miRNAs as potential GILZ regulators. Interestingly, there were more predictions for GILZ than for well-characterized miRNA targets, such as *CXCL8, IL6*, and *TNF* (34) (Figure 5F). To evaluate whether miRNAs are involved in Poly(I:C)-induced GILZ repression, AMs were either left untreated or treated with Poly(I:C), aurintricarboxylic acid (ATA), an inhibitor of ribonuclease activities which also affects miRNA processing (35), or a combination of both for 8 h, followed by quantification of GILZ protein levels. We observed that ATA treatment was able to prevent Poly(I:C)-mediated GILZ downregulation, suggesting that miRNAs indeed affect GILZ expression (Figure 5G).

**Figure 6:**
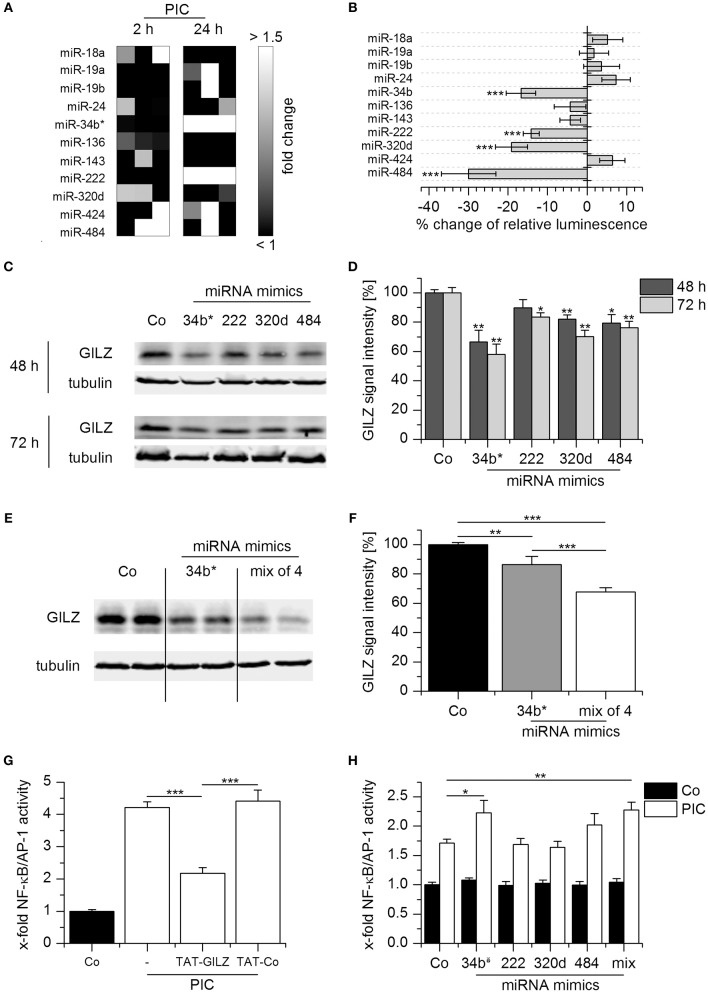
miRNA-mediated GILZ downregulation. **(A)** miRNA induction in Poly(I:C)-treated AMs (10 μg/mL for 2 h or 1 μg/mL for 24 h) was assessed by microarray analysis. Data are presented as fold change compared with untreated cells from the same donor. Individual changes per donor (*n* = 3) are shown. **(B)** The interaction of individual miRNAs with GILZ was determined by miRNA overexpression in HEKT293 cells co-transfected with a luciferase reporter construct containing the GILZ 3′UTR. Luminescence was measured 24 h after transfection and is shown as percent change compared with control vector-transfected cells lacking miRNA overexpression (*n* = 3–8, quintuplicates or sextuplicates). Significances were calculated in comparison with control vector-transfected cells. **(C,D)** HEK293T cells were transfected with miRNA mimics or scrambled controls (50 nM). GILZ expression was determined by Western blot at the indicated time points. **(C)** Representative blots. **(D)** GILZ signal intensities were quantified and normalized to tubulin values (*n* = 3, duplicates; Co: cells transfected with scrambled controls, set as 100%). **(E,F)** HEK293T cells were transfected with scrambled controls (50 nM, Co), miR-34b^*^ (12.5 nM miR-34b^*^ mimic + 37.5 nM scrambled controls), or a mix of miR-34b^*^, −222, −320d, and −484 mimics (12.5 nM each). GILZ expression was quantified after 48 h by Western blot. **(E)** Representative blot. **(F)** GILZ signal intensities were normalized to tubulin values (n = 3, duplicates or triplicates). Co values were set as 100%. **(G)** NF-kB/AP-1 activity was measured in untreated HEK-Blue reporter cells (Co) or after activation with PIC (1 μg/mL, 24 h) in the presence or absence of a cell-permeable GILZ peptide (TAT-GILZ, 2 μg/mL) or the respective control peptide (TAT-Co, 2 μg/mL; n = 3, triplicates). **(H)** HEK-Blue reporter cells were transfected with scrambled control (Co) or the indicated miRNA mimic. Twenty hours after transfection, cells were either left untreated or treated with PIC (1 μg/mL, 24 h), and NF-kB/AP-1 activity was determined (*n* = 3, triplicates). ^*^p < 0.05, ^**^p < 0.01, ^***^p < 0.001 vs. Co or as indicated.

Microarray analysis revealed that the expression of several potentially GILZ-targeting miRNAs was increased in Poly(I:C) treated AMs, although these effects were in some cases rather donor-dependent (Figure 6A). We tested the ability of 11 of these miRNAs predicted to target GILZ and expressed by Poly(I:C)-treated AMs to regulate GILZ by luciferase reporter gene assays. Within this small set, four miRNAs decreased the activity of the luciferase reporter encoded by a gene fused to the *GILZ* 3′UTR (Figure 6B). To determine the influence of these miRNAs on endogenous GILZ expression, we transfected HEK293T cells with mimics of the four candidate miRNAs, i.e., miR-34b^*^,−222,−320d, and−484, and observed that all mimics were able to reduce GILZ expression, although to a different extent (Figures 6C,D). To examine whether these miRNAs exert synergistic effects, HEK293T cells were transfected with the most potent mimic, i.e., miR-34b^*^, or a mix of all four miRNA mimics. As shown in Figures 6E,F, repression of GILZ expression was more efficient when using the miRNA mimic mix, suggesting that GILZ downregulation upon TLR3 activation is a consequence of the synergistic actions of multiple miRNAs.

We then wondered whether reduced GILZ expression upon miRNA mimic treatment might affect downstream effectors. To this end, HEK-Blue cells expressing a reporter gene under the control of the transcription factors NF-κB and AP-1 were used. Since HEK-Blue cells express endogenous TLR3, reporter gene activity was inducible by Poly(I:C). This effect was profoundly impaired by co-treatment with a cell-permeable GILZ peptide, indicating that GILZ can inhibit NF-κB/AP-1 activation in this system (Figure 6G). In line with these findings, Poly(I:C)-induced NF-κB/AP-1 activity was enhanced in HEK Blue cells transfected with the miR-34b^*^ mimic or a mix of all four mimics, i.e., under conditions that resulted in the highest degree of GILZ repression (Figure 6H).

## Discussion

To date, only a few studies have addressed the regulation of endogenous GILZ during the immune response in the absence of glucocorticoids. In both epithelial (36) and endothelial cells (37), inflammatory cytokines have been demonstrated to attenuate GILZ levels. GILZ expression has also been shown to be decreased in activated macrophages from patients with Crohn's disease or tuberculosis (6), and nasal explants from patients suffering from chronic rhinosinusitis lack GILZ (38). Furthermore, reduced *GILZ* mRNA levels were observed in liver tissue from patients with alcoholic hepatitis (17).

Besides the high abundance of GILZ in endothelial cells (21, 37), we previously demonstrated high GILZ levels in primary human monocyte-derived and pulmonary macrophages (21). GILZ expression was diminished in human alveolar macrophages as well as *in vivo* in mouse lungs upon TLR4 activation.

Further investigations indicated that *GILZ* mRNA was destabilized upon MyD88-mediated TLR activation, since LPS and Pam_3_CSK_4_, but not Poly(I:C), the activator of MyD88-independent TLR3, reduced *GILZ* mRNA expression. Downregulation of *GILZ* on the mRNA level required the presence of both the RNA-binding protein TTP and the *GILZ* 3'-untranslated region (21).

Our results presented within this study suggest that NF-κB activation is also required, which might at least in part be due to NF-κB-mediated TTP induction. Indeed, NF-κB has been previously shown to regulate TTP on the transcriptional level in LPS-stimulated RAW264.7 macrophages (39). However, the composition of the entire RNA-protein complex rather than the binding of TTP alone might dictate the fate of *GILZ* mRNA. The regulatory networks driving mRNA decay are considered to consist of multiple RNA-binding proteins, whose binding activity may be further modulated by miRNAs (40, 41). Thus, we cannot rule out the possibility that TTP-mediated *GILZ* mRNA destabilization requires accessory factors that may also be regulated by NF-κB.

Although Poly(I:C) did not decrease *GILZ* mRNA levels in macrophages, GILZ protein expression was reduced. Furthermore, Poly(I:C)-induced GILZ downregulation was dependent on both NF-κB activation and the presence of miRNAs. Our investigations revealed that multiple miRNAs act as GILZ regulators, suggesting that GILZ downregulation upon TLR3 activation is instead a consequence of synergistic miRNA actions. Promoter analysis using the miRGen v3.0 database (42) revealed that only miR-222 might be a direct target of NF-κB, whereas the promoters of miR-34b, miR-320d-1, and miR-484 lack NF-κB binding sites. NF-κB activation might influence miRNA expression indirectly, e.g., by modulation of miRNA processing via induction of the RNase Dicer (43). Finally, it is also possible that additional miRNAs are induced that were not identified by our approach.

Investigations on the functional significance of GILZ downregulation revealed an enhanced sensitivity toward LPS in human macrophages after siRNA-mediated GILZ knockdown, as indicated by increased cytokine expression and NF-κB activity (21). Within a follow-up study (11) we observed an increased response toward LPS in GILZ KO BMMs, such as enhanced NF-κB and AP-1 activity, suggesting repression of GILZ expression as a regulatory mechanism that amplifies macrophage activation. These findings are in line with our observation that treatment with a cell-permeable GILZ fusion protein can prevent PIC-induced NF-κB/AP-1-dependent activation in HEK-Blue cells.

In addition to its direct interaction with pro-inflammatory transcription factors, GILZ has also been reported to attenuate MAPK signaling in general and ERK signaling in particular (9–11). In the present study, we showed that the absence of GILZ increased both TLR1/2 and TLR3 activation, as indicated by augmented ERK activity, and, as a consequence, increased production of TNF-α. These findings suggest a role for GILZ repression in innate immune memory/trained immunity. Both terms describe an adaption of innate immune cell functions due to previous pathogen exposure, resulting in cross-protection between infections with different pathogens (32, 44).

The increased production of cytokines, such as TNF-α, promotes an acute inflammatory response that helps to clear invading organisms but also contributes to tissue damage. Another factor that plays a similarly ambivalent role in response to infections is nitric oxide (NO), a molecule with antimicrobial and proinflammatory functions. NO is generated from arginine in a reaction catalyzed by the enzyme nitric oxide synthase (NOS). Due to its inducible expression the principal isoform expressed in macrophages, i.e., NOS2, is also known as iNOS. iNOS expression can be elevated by activating cytokines, such as IFN-γ, and repressed by anti-inflammatory mediators, e.g., IL-4 or IL-10. Various PAMPs of bacterial, viral, or fungal origin have also been reported to enhance iNOS expression and function, with LPS as the most prominent example. LPS usually synergizes with IFN-γ to activate both NF-κB and STAT1, thereby inducing high levels of iNOS (45). Our data show that LPS- as well as LPS/IFN-γ-induced NO production can be increased by GILZ depletion. This finding is in line with the observation that LPS-induced NF-κB activity is increased in GILZ KO macrophages (11). In addition, we showed that treatment with TLR2 ligands, i.e., Pam_3_CSK_4_ and LTA, results in similar effects when co-administered with IFN-γ. TLR2 ligands did not, however, induce NO on their own, which might be attributed to the lack of TRIF-dependent IFN-β production and subsequent STAT1 activation upon TLR2 activation (46).

The ability to engulf particles, including pathogens and apoptotic cells, is a significant characteristic of macrophages. The phagocytic capacity is not necessarily associated with a specific macrophage phenotype, but rather constitutes a general attribute of these cells (24, 47). In this study, we show that lack of GILZ promotes phagocytosis in untreated as well as differentially polarized macrophages, including GC-treated cells. GCs have only recently been shown to impair phagocytosis by inhibiting the expression of genes that are required for phagosome formation (48). Whether or not these genes, such as *CD14, CD48*, and *MARCKS* are regulated in a GILZ-dependent manner presently remains elusive.

Although GILZ knockout macrophages showed a higher phagocytic activity, the number of viable bacteria in *S. typhimurium*-infected cells was reduced. This finding suggests that GILZ depletion enhances the bactericidal activity of macrophages, which is also supported by the observation that NO production is increased in GILZ knockout macrophages.

In conclusion, our data show that GILZ expression can be downregulated on different levels depending on the nature of the stimulus. In return, GILZ downregulation can promote essential macrophage functions, such as cytokine production, phagocytosis, and bactericidal activity. Thus, GILZ repression can help to clear pathogens, but might also be detrimental to the host by promoting excessive inflammatory disorders. Therefore, GILZ might represent a potential target for therapeutic interventions, both in order to restrict or to stimulate innate immune responses depending on the nature of the disease.

## Author Contributions

JH, BD, RL, NH, SF, MM, PL, and CB designed, performed, and analyzed the experiments. JH wrote the paper. All authors contributed to drafting the manuscript. SB, CR, FG, EM, and HH provided materials and discussed the data. AK initiated the study and participated in data interpretation and manuscript preparation. All authors read and approved the final manuscript.

### Conflict of Interest Statement

The authors declare that the research was conducted in the absence of any commercial or financial relationships that could be construed as a potential conflict of interest.

## Abbreviations

AMs: alveolar macrophages
AP-1: activator protein-1
ATA: aurintricarboxylic acid
BMMs: bone marrow-derived macrophages
CFU: colony-forming units
CXCL: C–X–C motif ligand
DMEM: Dulbecco's modified Eagle medium
ERK: extracellular signal regulated kinase
FCS: fetal calf serum
GC: glucocorticoid
GILZ: glucocorticoid-induced leucine zipper
HEK: human embryonic kidney
HMDMs: human monocyte-derived macrophages
GM-CSF: granulocyte macrophage colony stimulating factor
IL: interleukin
INF: interferon
iNOS: inducible NO synthase
KO: knockout
LPS: lipopolysaccharide
LTA: lipoteichoic acid
MOI: multiplicity of infection
NF-κB: nuclear factor-κB
MAPK: mitogen-activated protein kinase
M-CSF: macrophage colony stimulating factor
MPI: Max Planck Institute
MyD88: myeloid differentiation primary response gene 88
NO: nitric oxide
NOD: nucleotide-binding oligomerization domain
PMA: phorbol 12-myristate 13-acetate
Poly(I:C): polyinosinic:polycytidylic acid
PRR: pattern recognition receptor
RPMI: Roswell Park Memorial Institute
ROS: reactive oxygen species
RIG-1: retinoic acid-inducible gene-I
STAT: signal transducer and activator of transcription
TAT: trans-activator of transcription
TNF: tumor necrosis factor
TLR: toll-like receptor
TRIF: TIR domain-containing adapter inducing IFN-β
TTP: tristetraprolin
UTR: untranslated region
WT: wild-type.
