# Supplementary material for: Amplified Host Defense by Toll-Like Receptor-Mediated Downregulation of the Glucocorticoid-Induced Leucine Zipper (GILZ) in Macrophages
Source: Front Immunol. 2019 Jan 22;9:3111. doi: 10.3389/fimmu.2018.03111 (PMC6349698; doi:10.3389/fimmu.2018.03111)
Supplement: Supplementary file 1 [file Data_Sheet_1.pdf]

Supplementary Material

Amplified host defense by Toll-like receptor-mediated downregulation of the glucocorticoid-induced leucine zipper (GILZ) in macrophages

Jessica Hoppstädter[1]*, Britta Diesel[1], Rebecca Linnenberger[1], Nina Hachenthal[1], Sara Flamini[2], Marie Minet[1], Petra Leidinger[3], Christina Backes[4], Friedrich Grässer[5], Eckart Meese[3], Stefano Bruscoli[2], Carlo Riccardi[2], Hanno Huwer[6], Alexandra K. Kiemer[1]

[1] Pharmaceutical Biology, Department of Pharmacy, Saarland University, Saarbrücken, Germany

[2] Pharmacology, Department of Medicine, Perugia University, Perugia, Italy

[3] Human Genetics, Department of Medicine, Saarland University, Homburg, Germany

[4] Chair for Clinical Bioinformatics, Saarland University, Saarbrücken, Germany

[5] Virology, Department of Medicine, Saarland University, Homburg, Germany

[6] Cardiothoracic Surgery, Völklingen Heart Centre, Völklingen, Germany

*** Correspondence:**
Jessica Hoppstädter, Ph.D.
j.hoppstaedter@mx.uni-saarland.de

Supplementary Figures

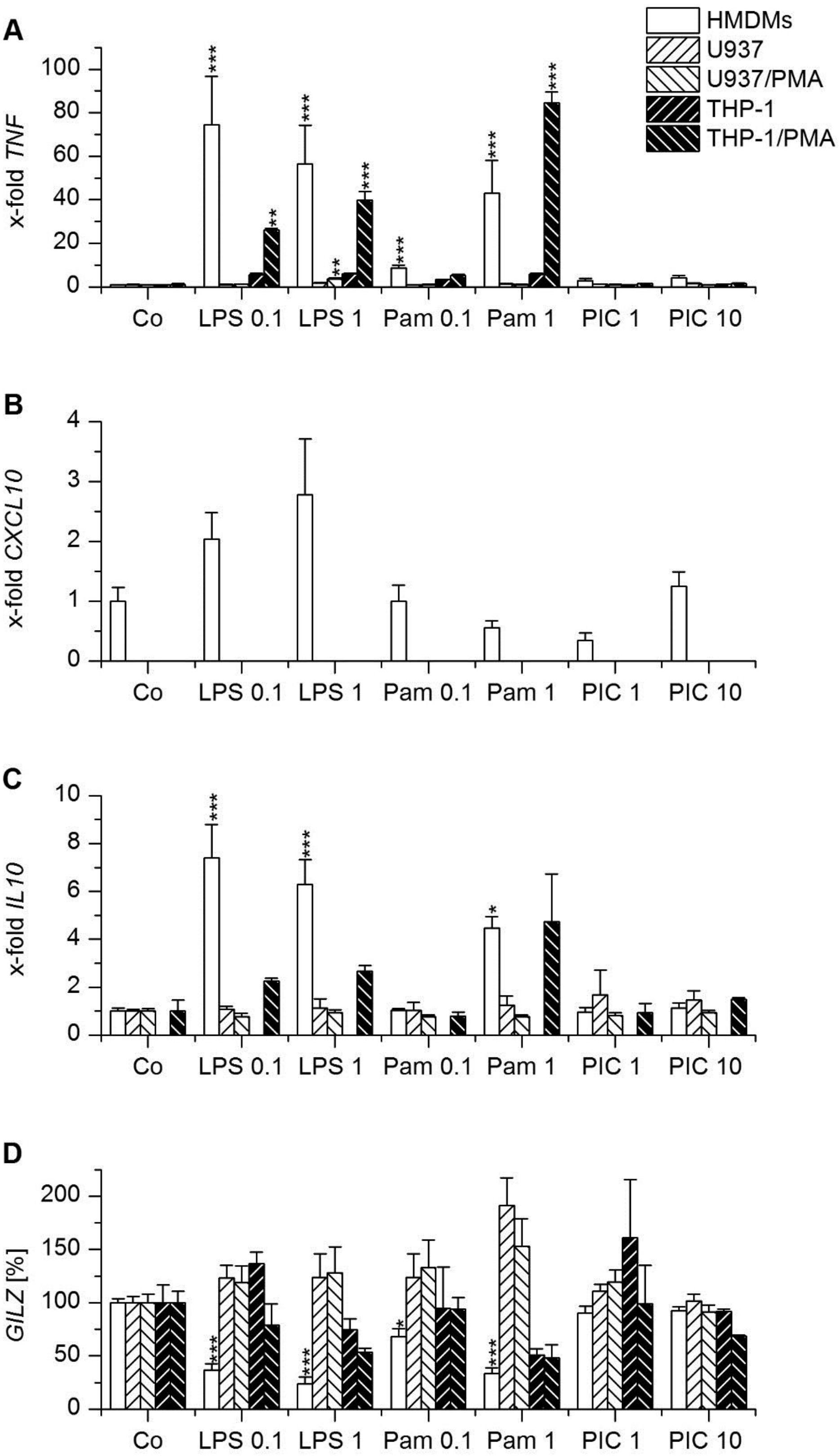

Supplementary Figure 1. *Responsiveness of human macrophages and macrophage-like cells towards the TLR1/2 ligand Pam$_3$CSK$_4$ and the TLR3 ligand Poly(I:C).* Human monocyte-derived macrophages (HMDMs), as well as undifferentiated or PMA-differentiated U937 and THP-1 cells were left untreated (Co) or treated with LPS (0.1 or 1 µg/ml), Pam$_3$CSK$_4$ (Pam, 0.1 and 1 µg/ml), or PIC (PIC, 1 and 10 µg/ml) for 2 h. and mRNA was quantified by qRT-PCR using *ACTB* as a housekeeping gene (n = 2-3, triplicates). *$p < 0.05$, **$p < 0.01$, ***$p < 0.001$ *vs.* Co.

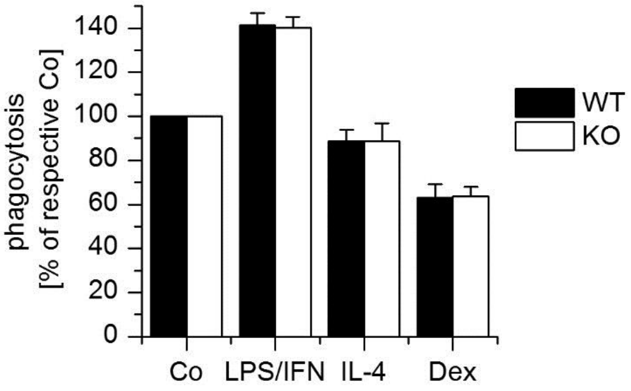

Supplementary Figure 2. *Different treatment schemes do not alter the effect of GILZ depletion on phagocytosis.* WT and GILZ KO BMMs were left untreated (Co) or treated with LPS (1 µg/ml) and INF-γ (IFN, 20 ng/ml), IL-4 (20 ng/ml) or dexamethasone (Dex, 1 µM) for 20 h, followed by incubation with fluorescent latex particles (diameter 1.75 µm, 50 particles per cell) for 1 h. Particle uptake was quantified by flow cytometry. The total percentage of cells with particle-associated fluorescence in LPS/IFN, IL-4, od Dex-treated cells was normalized to the respective value for cells that were not treated otherwise prior to particle exposure.

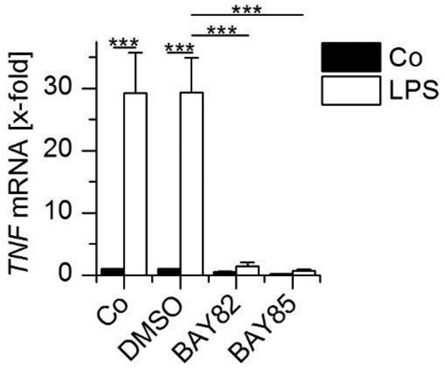

Supplementary Figure 3. *NF-κB inhibition.* After preincubation with BAY-11-7082 or BAY-11-7085 (5 µM, 1 h), solvent (0.1% DMSO) or medium only (Co), AMs were treated with LPS (100 ng/ml) for 2 h. *TNF* mRNA expression was determined by qRT-PCR using ACTB as a housekeeping gene (n = 3, duplicates).

Supplementary Tables

Supplementary Table 1: PCR conditions.

Gene	Sequence (5'→3') forward primer	Sequence (5'→3') reverse primer	Probe sequence (5'FAM→3'BHQ)	Probe [nM]	$MgCl_2$ [mM]	Annealing [°C]
IL6	AAT AAT AAT GGA AAG TGG CTA TGC	AAT GCC ATT TAT TGG TAT AAA AAC	TCC TTT GTT TCA GAG CCA GAT CAT TTC T	100	4	57
**IL8 (*CXCL8*)**	TGC CAG TGA AAC TTC AAG CA	ATT GCA TCT GGC AAC CCT AC	CAG ACC CAC ACA ATA CAT GAA GTG TTG A	100	4	59
TNF	CTC CAC CCA TGT GCT CCT CA	CTC TGG CAG GGG CTC TTG AT	CAC CAT CAG CCG CAT CGC CGT CTC	100	3	60
**GILZ (*TSC22D3*)**	TCC TGT CTG AGC CCT GAA GAG	AGC CAC TTA CAC CGC AGA AC	TCC CGA ATC CCC ACA AGT GCC CGA	100	4	60
**β-actin (*ACTB*)**	TGC GTG ACA TTA AGG AGA AG	GTC AGG CAG CTC GTA GCT CT	CAC GGC TGC TTC CAG CTC CTC	60	4	60
CXCL10	GAG CCT ACA GCA GAG GAA CC	AAG GCA GCA AAT CAG AAT CG	TCC AGT CTC AGC ACC ATG AAT CAA A	60	4	60
IL10	CAA CAG AAG CTT CCA TTC CA	AGC AGT TAG GAA GCC CCA AG	AGC CTG ACC ACG CTT TCT AGC TGT TGA G	100	4	60
**TTP (*ZFP36*)**	TCG CCA CCC CAA ATA CAA	TTC GCT AGG GTT GTG GAT	no probe	N/A	N/A	60

Supplementary Table 2: Primers used to clone miRNA overexpression vectors.

**miRNA**	**forward primer (plus EcoR1 site)**	**reverse primer (plus BamH1 or BglII site)**
hsa-miR-18a	GC GAA TTC ATG GGA AGC CAA GTT GGG CTT TAA	GA GGA TCC CAC CTA TAT ACT TGC TTG GCT
hsa-miR-19a	GC GAA TTC ATG ATC CAA TAA TTC AAG CCA AGC	GA GGA TCC GCA GAT TCT ACA TCG ACA CAA
hsa-miR-19b	GC GAA TTC ATG TAG CTG TAG AAC TCC AGC TTC	GA GGA TCC GGG TTT GAG TTT CCC TTA CTT
hsa-miR-34b	GC GAA TTC ATG TAC GCG TGT TGT GCG CTG CGA	GA GGA TCC AAC CGC GGG TTT CCT CGC ACT
hsa-miR-136	GC GAA TTC ATG AGC TCT TCC ATT TCC TGG AGT	GA GGA TCC TCT GCT CTG ATT AGT TGG GCA
hsa-miR-222	GC GAA TTC ATG GAA AAT ATG TGG CAC TTT ATT	GA GGA TCC CTT AAC ACC CTA GAA CTT GAC
hsa-miR-320d1	GC GAA TTC ATG CCT TCA AAT GAC AAA ACA CAC	GA GGA TCC TTC CTT TCC TAT TTC TTT CCT
hsa-miR-484	GC GAA TTC ATG AAA ACC GAC GCC CTT CTC TCC	GA GGA TCC GTC CAC GTC ACG AGC TCA TTC
